# Adding energy gradients and long‐distance dispersal to a neutral model improves predictions of Madagascan bird diversity

**DOI:** 10.1002/ece3.2379

**Published:** 2016-09-07

**Authors:** Falko T. Buschke, Luc Brendonck, Bram Vanschoenwinkel

**Affiliations:** ^1^ Centre for Environmental Management (67) University of the Free State P.O. Box 339 Bloemfontein 9300 South Africa; ^2^ Laboratory of Aquatic Ecology, Evolution and Conservation KU Leuven Ch Deberiotstraat 32 3000 Leuven Belgium; ^3^ Department of Biology Vrije Universiteit Brussel Pleinlaan 2 1050 Brussels Belgium

**Keywords:** Birds, dispersal, drift, Madagascar, mid‐domain effects, neutral theory, stochastic

## Abstract

Macroecological patterns are likely the result of both stochastically neutral mechanisms and deterministic differences between species. In Madagascar, the simplest stochastically neutral hypothesis – the mid‐domain effects (MDE) hypothesis – has already been rejected. However, rejecting the MDE hypothesis does not necessarily refute the existence of all other neutral mechanisms. Here, we test whether adding complexity to a basic neutral model improves predictions of biodiversity patterns. The simplest MDE model assumes that: (1) species' ranges are continuous and unfragmented, (2) are randomly located throughout the landscape, and (3) can be stacked independently and indefinitely. We designed a simulation based on neutral theory that allowed us to weaken each of these assumptions incrementally by adjusting the habitat capacity as well as the likelihood of short‐ and long‐distance dispersal. Simulated outputs were compared to four empirical patterns of bird diversity: the frequency distributions of species richness and range size, the within‐island latitudinal diversity gradient, and the distance‐decay of species compositional similarity. Neutral models emulated empirical diversity patterns for Madagascan birds accurately. The frequency distribution of range size, latitudinal diversity gradient, and the distance‐decay of species compositional similarity could be attributed to stochastic long‐distance migration events and zero‐sum population dynamics. However, heterogenous environmental gradients improved predictions of the frequency distribution of species richness. Patterns of bird diversity in Madagascar can broadly be attributed to stochastic long‐distance migration events and zero‐sum population dynamics. This implies that rejecting simple hypotheses, such as MDE, does not serve as evidence against stochastic processes in general. However, environmental gradients were necessary to explain patterns of species richness and deterministic differences between species are probably important for explaining the distributions of narrow‐range and endemic species.

## Introduction

Macroecological patterns in nature are caused by combinations of stochastically neutral processes and deterministic differences between species' niches (Gravel et al. 2006). However, it is often more practical to make simplifying assumptions to theories so that they no longer depict natural phenomena precisely, but rather approximate several different patterns using few free parameters (Marquet et al. 2014). This means that most studies focus primarily on either stochastically neutral models or deterministic models of niche‐based differences (but for exceptions, see Gravel et al. 2006; Latombe et al. 2015). In highly diverse systems where deterministic differences between individual species are difficult to measure, it is simpler to assume equivalence between species in order to model otherwise intractable problems (Rosindell et al. 2012).

In Madagascar, the validity of stochastically neutral processes has been challenged during recent debates on the mid‐domain effects (MDE) hypothesis. Madagascar switched from being one of the most convincing cases of MDE (Colwell et al. 2004; Pimm and Brown 2004) to a classical counter‐example (Kerr et al. 2006; Currie and Kerr 2008). MDE predict that gradients of species richness may arise “even without environmental gradients” (Colwell and Lees 2000) from the stochastic and nonbiological arrangement of species' ranges (Colwell and Hurtt 1994). Continuous (*i.e.,* unfragmented) species ranges are more likely to overlap at the centre of a bounded landscape by chance alone. Early research showed how the diversity of forest species in Madagascar tended to peak at the centre of the biome, despite environmental gradients varying monotonically with latitude (Lees et al. 1999). However, Kerr et al. (2006) later demonstrated how this pattern disappeared once they accounted for the propensity of latitudinal bands nearer the middle of the island to have larger surface areas. Moreover, the MDE identified by Lees et al. (1999) may have been caused by erroneous interpolation of species occurrences into under‐sampled areas (Currie and Kerr 2008). Although reanalyses by Lees and Colwell (2007) confirmed that the mid‐latitude peak in species richness within the eastern moist forests of Madagascar did indeed conform to MDE predictions, Currie and Kerr (2007) rightfully queried the generality of this pattern due its absence from dry forest and grassland habitats.

The MDE hypothesis in Madagascar mirrors its status elsewhere. Early evidence supported the hypothesis in New World bats and marsupials (Willig and Lyons 1998) as well as in birds of Africa (Jetz and Rahbek 2001) and the New World (Romdal et al. 2005). However, Currie and Kerr (2008) found in their comprehensive review that 50 of 53 studies published up to that point demonstrated significant deviations from MDE predictions. Moreover, the few cases consistent with MDE had collinear environmental gradients to explain the patterns (Currie and Kerr 2008). This suggests that species respond differently to environmental conditions, and it is these deterministic processes that determine patterns of diversity. In this study, we investigate whether the failure of the MDE hypothesis should imply the failure of stochastic and neutral processes in general. We do this by simulating diversity patterns for Madagascan birds using a model based on neutral theory (Hubbell 2001), which is still ecologically neutral and stochastic, but is based on more realistic assumptions.

Although there is broad consensus that MDE only provide weak explanations of diversity patterns in nature (Zapata et al. 2003; Currie and Kerr 2008), explicit tests for why this is so are lacking. This may be because the MDE hypothesis, as it was originally presented, contained no explicit mechanism to account for the spatial cohesion and limits of species ranges (Gotelli and McGill 2006). Instead, it has been implicitly assumed that the failure of MDE was caused by deterministic processes associated with environmental gradients. However, stochastic mechanisms might provide equally valid explanations for the weak support for the MDE hypothesis. First, MDE assume that species' ranges are continuous and unfragmented, which will only be true if communities are exclusively linked by short‐distance dispersal (Rangel and Diniz‐Fiho 2005). Random long‐distance dispersal can, therefore, account for patterns inconsistent with MDE without evoking any deterministic processes. Second, MDE assume that the frequency distribution of species' range area is a fixed pattern that remains unchanged, regardless of the position of the individual ranges. It was originally argued that in the absence of deterministic environmental gradients, all species would be equally widespread and uniformly distributed across the domain (Hawkins and Diniz‐Filho 2002; Zapata et al. 2003). However, more recent studies show that stochastic mechanisms may also give rise to realistic range size frequency distributions (Rangel and Diniz‐Fiho 2005; Keith and Connolly 2013). Lastly, MDE requires that the placement of species ranges in a domain is random and, therefore, independent of other species or environmental gradients. This is a weak assumption because energetic constraints are elementary to ecology (Hurlbert and Stegen 2014; Rabosky and Hurlbert 2015). As such, incorporating diversity‐dependent range dynamics into simulations (*i.e.,* limiting range expansion into sites that already contain many other species) drastically weakens any MDE because ranges can no longer be stacked indefinitely (Keith and Connolly 2013). This implies that uniform energetic constrains, rather than deterministic species' responses to environmental gradients, could account for diversity patterns in nature.

Our study uses simulations based on neutral theory to test the degree to which the diversity of birds in Madagascar can be attributed to stochastic mechanisms. Specifically, we examine whether incorporating directional long‐distance dispersal improves the predictive ability of stochastic mechanisms. We then examine whether these same stochastic processes can emulate empirical range size frequency distributions, which have previously been offered as evidence for environmental determinism (Hawkins and Diniz‐Filho 2002; Zapata et al. 2003). Finally, we examine whether diversity patterns can be explained by uniform density‐dependent dynamics (*e.g.,* Keith and Connolly 2013), or whether they rely on gradients in the energetic constraints that limit the number of organism that can occur at a locality.

## Methods

### Bird species distribution data

Bird species distribution data, expressed as the extent of occurrence, were obtained from Bird Species Distributions Maps of the World version 1.0 (BirdLife International & NatureServe, 2011). Complete spatial data were only available for 224 of the approximately 265 species ever observed in Madagascar (Morris and Hawkins 1998), which included data for 112 species endemic to Madagascar and the surrounding islands. We excluded marine birds, rare vagrants, and species that are globally or regionally extinct. We covered the island with a regular grid of 280 equal‐area quadrats measuring 50 km by 50 km using the Tananarive 1925 (UTM zone 38S) projected coordinate system and assigned species to quadrats if any part of their distribution ranges coincided with the quadrats.

We expected that a neutral model within Madagascar might explain diversity for endemic and nonendemic birds with varying success. In Madagascar, nonendemic birds typically dispersed from Africa (Yoder and Nowak 2006), which suggests that immigration was consistently more prevalent at the western coastline than the east. In contrast, evidence for MDE – and, therefore, stochastic mechanisms – is expected to be stronger in endemic species than for nonendemics (Colwell and Lees 2000). We, therefore, repeated all subsequent analyses of the entire bird assemblage (because energetic constraints will influence the entire assemblage simultaneously) in Madagascar as well as for the subset of endemic species separately.

For both the whole assemblage and the subset of endemics, we quantified four diversity patterns: (1) the frequency distribution of species richness, (2) the latitudinal diversity gradient, (3) the rank‐occupancy curve, and (4) the distance‐decay of species compositional similarity. Species richness was quantified as the number of ranges within a quadrat. The latitudinal diversity gradient cumulated the number of species occurring in horizontal 50 km bands, but unlike Kerr et al. (2006) we did not add a correction for the land surface area within these bands. While they were interested in the specific form of the latitudinal diversity gradients (as a test for MDE), we simply wanted to investigate whether empirical patterns were matched by those simulated by the neutral model. Latitudinal band areas were identical for both empirical and simulated patterns, so correcting for area would have no effect on our analyses because it divides both the empirical and simulated data by the same denominator without changing their association. The rank‐occupancy curve is a way to visualize the range size frequency distribution without assigning range sizes – quantified as the number of occupied quadrats – to frequency classes. Instead, it plots the occupancy of a species against the rank of its occupancy. Lastly, to quantify species compositional similarity, we used pair‐wise comparisons of the Jaccard's similarity index (Jaccard 1912), which is the number of species shared by two quadrats divided by the combined number of species in both quadrats. To determine the average distance‐decay of similarity for the whole island, we calculated the mean and standard deviation of Jaccard similarities within 25 km distance classes.

### Simulating diversity patterns under neutral dynamics

It is common in macroecology to construct a simple model with very basic assumptions to which varying levels of complexity can be added (Gotelli et al. 2009). However, this approach did not suit our neutral model, which required specific parameterization. Instead, we started by constructing and parameterizing the most complex model that included gradients in energetic constraints as well as both short‐ and long‐distance dispersal. We then either sequentially removed one of these aspects (*i.e*., energy gradients) from the model or varied the parameters continuously (*i.e*., Habitat capacity, *K*, short‐, *a*, and long‐distance, *b*, dispersal) in order to evaluate their importance in explaining diversity patterns for Madagascan birds.

Our neutral model was inspired by that of Muneepeerakul et al. (2008), which simulated diversity patterns for fish in the Mississippi–Missouri River basin. We simulated two separate models in R version 3.1.2 (R Core Team, 2014): one for all 224 bird species and one only for 112 endemic bird species. Our model simulated the dynamics of “bird units,” which can be seen as a subpopulation of birds of the same species with equivalent *per capita* relative fitness. An equivalent number of individuals within each bird unit will likely differ among species depending on their mating systems, age of maturation, clutch size, and level of parental care. The concept of a bird unit is similar to that of a “propagule,” which MacArthur and Wilson (1963) defined as “the minimum number of individuals of a given species needed to achieve colonization.” The number of bird units in each quadrat *i*, regardless of species identity, was determined by the habitat capacity (*H*_*i*_) rounded up to the nearest integer.

$$H_{i}=K\times J\frac{{\text{NPP}}_{i}}{\sum_{i=1}^{J}{\text{NPP}}_{i}}$$

Here, *K* is a constant free parameter for the average habitat capacity of a quadrat, *J* is the total number of quadrats (*J *=* *280), and NPP_*i*_ is the net primary productivity of quadrat *i*. NPP was estimated from normalized difference vegetation index (NDVI) data, which were averaged across three measurements per month for the period 2000–2004 (Tucker et al. 2005). The product of *K* and *J* is the total number of bird units in the whole of Madagascar and *H*_*i*_ is directly proportional to NPP_*i*_. Incorporating NPP in our simulations added a gradient in the maximum number of bird units that can persist in a quadrat. To test the importance of this gradient in explaining diversity patterns, we contrasted the outputs from this simulation to those where all quadrats were assigned identical habitat capacities (*H*_*i*_ **= *K*).

To initiate the model, one bird unit for each species type (224 and 112 species types for all and endemic species, respectively) was randomly assigned to a quadrat. At each subsequent time step of the model, a bird unit was added to every one of the quadrats either by immigration from a neighboring quadrat or by a birth from within the quadrat. Unoccupied quadrats had to first be colonized before any birth process could occur within it. This probability that a bird unit from quadrat *j* would be added to quadrat *i* was quantified as

$$I_{ij}=\frac{R_{ij}\times H_{j}}{\sum_{k=1}^{J}R_{ik}\times H_{k}}$$

where *R*_*ij*_ is the dispersal kernel (see below). This function declares that migration was more probable from quadrats with higher habitat capacities to those with lower habitat capacities. Again, we repeated the simulations after making dispersal equally likely regardless of the habitat capacity (*H*_*j*_ = *H*_*k*_ = 1) to quantify the effects of these directional source‐sink dynamics. The dispersal kernel, *R*_*ij*_, was expressed as

$$R_{ij}=C\left[a^{D_{ij}}+\frac{b^{2}}{D_{ij}^{2}+b^{2}}\right]$$

Here, the likelihood of dispersal between quadrats *i* and *j* was determined by the standardized geographic distance between the two quadrats, *D*_*ij*_. Distances between quadrat centroids were standardized by dividing the geographic distance in kilometers by 50, so adjacent quadrats were separated by 1 distance unit and quadrats 1000 km apart were separated by 20 distance units. Two constant free parameters, *a* and *b*, characterized the shape of the dispersal kernel and *C* is the normalization constant (Muneepeerakul et al. 2008). Short‐distance dispersal was varied by manipulating parameter *a* in the part of the dispersal kernel characterized by an exponential function. Contrastingly, the likelihood of long‐distance dispersal was varied by adjusting parameter *b* in the part of the dispersal kernel characterized by a Cauchy function.

At each step of the simulation, a single birth/immigration event occurred across every one the quadrats simultaneously. For each quadrat, *i*, a bird unit was added after selecting the source quadrat of the new bird unit with a probability *I*_*ij*_. Then, a single bird unit was selected from the source quadrat with an equal probability so that the likelihood of selecting from a specific species was proportional only to the abundance of that species (*i.e.,* the number of bird units of that species). The selected bird unit was cloned and the clone was added to the focal quadrat (*i.e.,* the original unit remained behind in the source quadrat). This process meant that the number of bird units in each quadrat increased linearly with each step of the simulation. A mortality process was started as soon as the number of bird units in quadrat *i* reached the habitat capacity, *H*_*i*_. This mortality process simply selected a bird unit at random and removed it from a quadrat prior to adding the new bird unit. Again, the likelihood of any individual bird unit being selected for mortality was equiprobable, but the probability of a species being selected was proportional to its abundance.

### Parameterizing the neutral model

Our simulation had three free parameters: the average number of bird units per quadrat, *K*, and the likelihood of short‐, *a*, and long‐distance, *b*, dispersal. We examined several combinations of these three parameters to minimize the difference between the simulated outcomes and the empirical patterns. It should be emphasized; however, that there is not necessarily a best fitting set of free parameters because simulations capture the outcomes of stochastic and nonlinear processes. Instead, there is probably a broad space of parameters that replicate empirical patterns equally well (*e.g*., Etienne et al. 2006). Furthermore, the suitability of a set of parameters also depends on the length of the simulation (the number of time steps) because it models dynamic processes that vary with time. We found that the model generally stabilized after approximately 40,000 time steps, but destabilized again after approximately 100,000 time steps (Appendix S1). This destabilization is likely due to local extinctions caused by random drift (sensu Halley and Iwasa 2011), which could not be offset by the formation of new species because our model did not include speciation (Appendix S2). As a consequence, we evaluated a set of parameters after running a simulation for 50,000 time steps.

For every set of parameters, we computed the error between the empirical and simulated patterns of species richness, latitudinal diversity, occupancy, and Jaccard similarity. We estimated the error of each of the four patterns as the mean square deviation between empirical and predicted data, normalized by the variance of the empirical data:

$$\text{Error}=\frac{\sum_{i=1}^{N}{\left(x_{i}-{\hat{x}}_{i}\right)}^{2}}{\sum_{i=1}^{N}{\left(x_{i}-〈x〉\right)}^{2}}$$

where *N* is the number of observations for that specific pattern. The empirical value of observation *i* is *x*_*i*_, the predicted value of observation *i* is ${\hat{x}}_{i}$, and $〈x〉$ is the mean of the empirical observations. The total error of the simulated model was the sum of the four independent errors for richness, latitudinal diversity, occupancy, and Jaccard similarity. We also obtained the *R* ^2^ for a unity line linear regression (Romdal et al. 2005) by subtracting the prediction error from one. The *R* ^2^ from the unity line regression quantifies how well the simulation predicted the empirical patterns compared to a straight line with a slope of 1 and an intercept of 0, which indicates perfect prediction. It must be noted, however, that the set of parameters with the lowest total error was not necessarily the same as to the set that minimized the error for each of the four diversity patterns independently (see for comparison the approach by May et al. 2015).

We first ran a general scan of the parameter space and then used incremental changes to parameters *K, a* and *b*, to minimize the total error. This was based on an iterative process from starting parameters *K* = 500, *a* = 0.2, *b* = 0.2. First, *K* was adjusted upwards and downwards in 100 unit increments to minimize total error. Next *a* and then *b* were sequentially adjusted in units of 0.05 to minimize total error. After identifying the best qualitative fit, we then examined the full parameter space between the two best fitting values for *K*,* a* and *b* by simulating all combinations of these parameters (*K* in increments of 50 and *a* and *b* in units of 0.005). Simulations for each set of parameters were run in duplicate, and the prediction error was averaged across both runs. Once we identified the set of parameters that minimized prediction error, we then carried out a sensitivity analysis by holding two of the parameters constant while varying the third incrementally. This allowed us to examine how sensitive the model predictions were to the choice of parameters.

## Results

**Figure 1:**
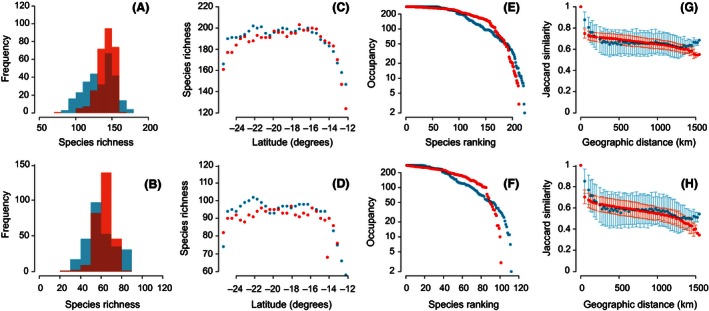
Empirical (blue) and simulated (red) frequency distribution of species richness (A, B), latitudinal diversity gradient (C, D), rank‐occupancy curve (E, F) and distance‐decay of community similarity (G, H) for all (A, C, E, G) and endemic (B, D, F, H) birds species in Madagascar. For the distance‐decay of similarity, G, H) points and error bars reflect the mean and standard deviation, respectively, of Jaccard similarity in 25 km distances classes. The simulation parameters minimized the total error (*K *=* *700, *a *=* *0.19, *b *=* *0.18 for all species and *K *=* *350, *a *=* *0.095, *b *=* *0.09 for endemic species).

Simulations based on neutral theory emulated empirical patterns for Madagascan birds with reasonable success (Fig. 1). When parameters were chosen to minimize total error (*K *=* *700, *a *=* *0.19, *b *=* *0.18 for all species and *K *=* *350, *a *=* *0.095, *b *=* *0.09 for endemic species), predictions for all species were more accurate than those for only endemic species, in terms of both total error and the error for each pattern separately (Table 1). Neutral simulations predicted the shape of the rank‐occupancy curve for all and endemic bird species with remarkable accuracy (Table 1; Fig. 1E and F), thereby countering claims that the range size frequency distribution is necessarily due to environmental gradients. However, the prediction accuracy for other patterns of diversity was more variable. Simulations consistently underestimated the variation in the frequency distribution of species richness (Fig. 1A and B), but had more success in predicting the richness in latitudinal bands (Fig. 1C and D). This suggests that neutral models can recreate the extent, but not necessarily the placement of species' ranges (Appendix S3). Simulation outputs matched empirical patterns of the distance‐decay of community similarity, although they tended to underestimate similarity at particularly short and long geographic distances (Fig. 1G and F).

**Table 1:** The error and *R* ^2^ from a unity line regression for the predictive relationship between diversity patterns generated by neutral simulations and those for all and endemic birds in Madagascar. The simulation parameters used to minimize the total error were *K *=* *700, *a *=* *0.19, *b *=* *0.18 for all species and *K *=* *350, *a *=* *0.095, *b *=* *0.09 for endemic species

Diversity patterns	All species		Endemic species	
	Error	$R_{\mathrm{Unity}\;\mathrm{line}}^{2}$	Error	$R_{\mathrm{Unity}\;\mathrm{line}}^{2}$
Frequency distribution of species richness	0.78	0.22	1.15	0
Latitudinal diversity gradient	0.15	0.85	0.33	0.66
Rank‐occupancy curve	0.09	0.91	0.08	0.92
Distance‐decay of Jaccard similarity	0.48	0.52	0.46	0.54
Total error	1.49		2.03	

**Figure 2:**
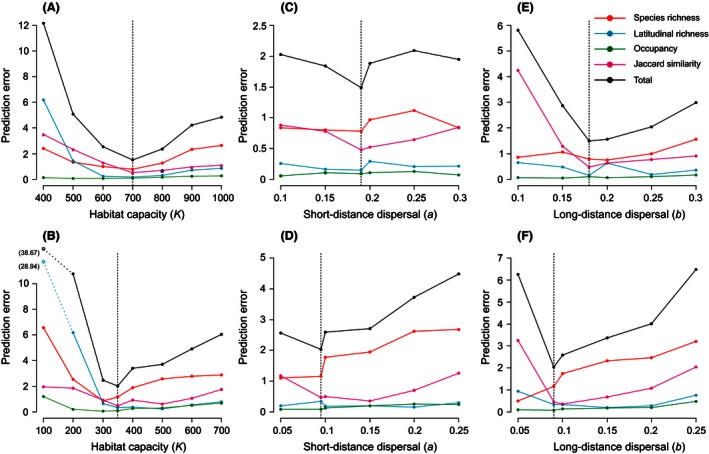
The sensitivity of prediction error from simulated neutral model to changes in parameters of habitat capacity (A, B), short‐distance dispersal (C, D) and long‐distance dispersal (E, F) for all (A, C, E) and endemic (B, D, F) birds species in Madagascar. Vertical dashed lines denote the parameter that minimized total prediction error.

Simulations suggested that diversity patterns for Madagascan birds are sensitive to the habitat capacity of each quadrat (Fig. 2A and B). This was especially true for the frequency distribution of species richness for all and endemic birds. The distance‐decay of Jaccard similarly seemed to be sensitive to changes in habitat capacity related to energetic constrains (Fig. 2A), as was the latitudinal diversity gradient for endemic birds (Fig. 2B). Remarkably, the rank‐occupancy curve for all and endemic birds was robust to changes in habitat capacity.

**Figure 3:**
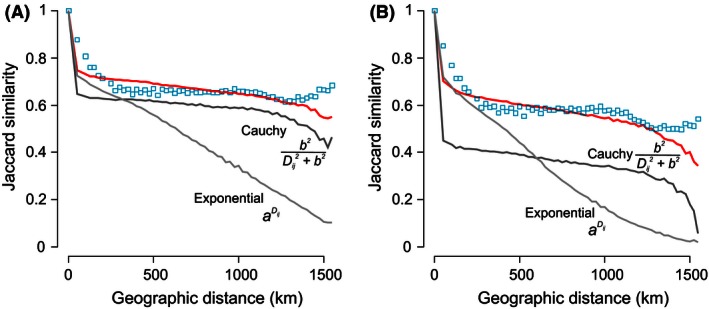
The effect of combining exponential and Cauchy functions in predicting the distance‐decay of community similarity of all (A) and endemic (B) birds in Madagascar. Blue squares indicate the mean empirical Jaccard similarity in 25 km distances classes. Red lines represented the mean Jaccard similarity in 25 km distance classes simulated by a neutral model using a composite dispersal kernel of exponential and Cauchy distributions (parameters *a* and *b* as used in Fig. 1). Gray lines show the predicted Jaccard similarity from simulations using dispersal kernels that only include exponential (light gray; *b *=* *0) or Cauchy (dark gray; *a *=* *0) distributions.

Both latitudinal diversity and rank‐occupancy for all and endemic birds were robust to changes in the parameter associated with short‐distance dispersal, *a*, whereas the distribution of richness and distance‐decay of Jaccard similarity were more sensitive to this parameter (Fig. 2C and D). This was also true for the parameter associated with long‐distance dispersal, *b*, because latitudinal diversity and rank‐occupancy seemed to be indifferent to changes in long‐distance dispersal (Fig. 2E and F). The frequency distribution of species richness was very sensitive to increased long‐distance dispersal, whereas the distance‐decay of Jaccard similarity was disproportionally sensitive to a decrease in long‐distance dispersal (Fig. 2E and F). The distance‐decay of Jaccard similarity illustrated particularly well how both short‐ and long‐distance dispersal interact to emulated patterns of diversity (Fig. 3).

**Figure 4:**
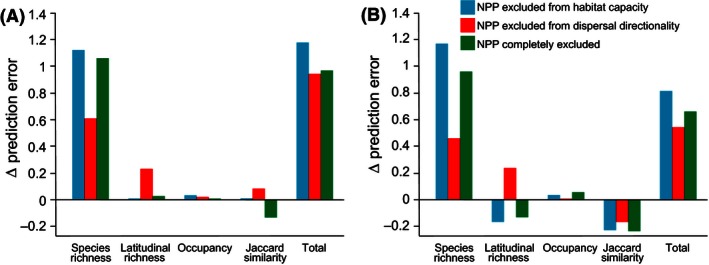
The change in prediction error from the neutral model for diversity patterns of all (A) and endemic (B) birds in Madagascar caused by removing the effects of net primary productivity (NPP) from habitat capacity (blue), dispersal directionality (red), or both habitat capacity and dispersal directionality (green).

Including a gradient in habitat capacity by adjusting energy constraints generally improved the ability of our simulations to predict empirical patterns (Fig. 4). However, the greatest effect of this gradient was in improving the prediction of the frequency distribution of species richness; probably because variations in species richness were directly related to habitat capacity (Appendix S4). Incorporating NPP to determine the habitat capacity and the directionality of dispersal had little to no effect on other patterns (Fig. 4).

## Discussion

Stochastic and neutral simulations can predict several key biodiversity patterns for Madagascan birds. This contrasts with the assumption that stochastic explanations of diversity patterns are negligible compared to deterministic species responses to environmental gradients. Deterministic processes in Madagascar gained support after the island was demoted from being an “impressive victory” for MDE (Pimm and Brown 2004) to being a classical counter‐example (Kerr et al. 2006; Currie and Kerr 2008). However, the results from our study illustrate the pitfalls of using the MDE hypothesis as an alternative hypothesis to deterministic environmental processes in general. Instead, it is also likely that the lack of evidence for the MDE hypothesis can be attributed to energetic constraints and long‐distance dispersal.

Our simulations do not necessarily imply that the empirical patterns in Madagascar are actually the consequence of neutral processes. Our simulations still suffer from the problem of coherence (Clark 2012) because we did not prove that species occupy the same ecological niches or have equivalent *per capita* fitness. Even so, we argue that neutral theory is not a test for the *existence* of deterministic niche‐based mechanisms, but rather whether such mechanisms are *detectable* in empirical data (Rosindell et al. 2012). Niche‐based and neutral processes may result in indistinguishable ecological patterns and may even amplify one another through feedbacks when they act concurrently (Latombe et al. 2015). As such, we take the position that niche‐theory should not be viewed as an alternative to neutral theory, but rather as an incremental extension of it (Gotelli and McGill 2006). Thus, neutral theory – along with assumptions of stochasticity and ecological equivalence – is a useful starting point for explaining contemporary patterns of Madagascan bird diversity because it makes multiple predictions that match several empirical patterns simultaneously (Marquet et al. 2014; May et al. 2015).

Unlike MDE, neutral theory is flexible and allowed us to adjust the habitat capacity and likelihood of short‐ and long‐distance dispersal. Long‐distance dispersal events have major consequences for biogeographical patterns (Gillespie et al. 2012), yet patterns consistent with MDE require that populations are only linked by short‐distance dispersal (Rangel and Diniz‐Fiho 2005). The dispersal kernel in our simulations was a composite of exponential and Cauchy functions, which balanced the probabilities of short‐ and long‐distance dispersal, respectively. This still constrained dispersal, but allowed for occasional long‐distance migration events, which is a reasonable assumption for vagile organisms such as birds. This adjustment to the dispersal kernel seems to be central to empirical patterns of diversity because simulation outcomes for both species richness and Jaccard similarity were particularly sensitive to changes in the short‐ and long‐distance dispersal. However, short‐distance dispersal was still much more probable than long‐distance dispersal in our models, so many species might not have experienced any long‐distance dispersal events during the duration of the simulation. As a consequence, it remains to be shown whether the complex dispersal kernel in our simulations demonstrates the significance of long‐distance dispersal in general, or whether it simply created variation in the dispersal of different species that more closely emulates the nonneutral dispersal found in nature (Lowe and McPeek 2014). Moreover, short‐ and long‐distance dispersal was more prevalent in the whole assemblage compared to endemic species. This is consistent with interisland dispersal limitation contributing to the generation of endemic species in Madagascar (Wilmé et al. 2006; Vences et al. 2009).

Overall, zero‐sum dynamics result in patterns vastly different to those from unconstrained (non‐zero‐sum) dynamics (Hurlbert and Stegen 2014; but see Etienne et al. 2007). Although we did not compare constrained and nonconstrained dynamics explicitly (sensu Keith and Connolly 2013), predictions from our simulations were sensitive to the parameter related to habitat capacity. This suggests that the model predictions are not only sensitive to the presence, but also to the degree, of energetic constrains. Furthermore, incorporating gradients in NPP generally improved the predictions from our simulations (Fig. 4); especially for patterns of species richness. Previous studies have found an almost ubiquitous relationship between NPP and species richness (Hawkins et al. 2003). Our study extends these findings by implying that species richness is not just due to a combination of sampling effects and reduced extinction rates associated with higher population sizes (More individuals hypothesis: Wright 1983; Clarke and Gaston 2006), but also because gradients in NPP may influence the directionality of dispersal. Results suggest that models match empirical patterns more closely when dispersal from high‐energy quadrats was more likely than from low‐energy quadrats (source‐sink dynamics). Although the effect of NPP on dispersal was smaller than the effect on habitat capacity, it offers a dispersal‐based explanation for the energy–diversity relationship to complement conventional mechanisms related to population size, niche‐position, and diversification rates (Evans et al. 2005).

Intriguingly, removing gradients in energetic constrains actually improved predictions of the distance‐decay of community similarity, albeit to a lesser degree than the negative effects on species richness. This may be because quadrats with higher NPP will not only contain more species, but are also more likely to be a source of migrants. As such, we speculate that environmental gradients increase the nestedness component of beta diversity (*i.e.,* species addition or removal) because species‐poor quadrats are more likely to be comprised of a subset of species from a species‐rich quadrat in the presence of energy gradients. In Madagascar, endemic species are expected to have higher turnover components of beta diversity (*i.e.,* species replacement) because their distributions are more likely caused by the radiation of neoendemics in separate centers of endemism than range expansion from a single source (Wilmé et al. 2006). This may explain why including gradients in NPP had a larger affect on predictions of the distance‐decay community similarity in endemic species compared to the entire assemblage.

Early critics of MDE argued that range size frequency distribution was the result of environmental gradients because species would occur everywhere unless their ranges were constrained by the environment (Hawkins and Diniz‐Filho 2002; Zapata et al. 2003). Our results counter such claims and, therefore, support studies demonstrating that realistic range size frequency distributions can arise from stochastic mechanisms (Rangel and Diniz‐Fiho 2005; Keith and Connolly 2013). The results from our study even go so far to suggest that the shape of the range size frequency distribution is indifferent to changes in underlying mechanisms in general. Sensitivity analysis across a range of parameters confirmed that model predictions for this specific pattern were robust to our choice of simulation parameters. However, even though our neutral model could predict the frequency distribution of range sizes, it struggled to predict the placement of these ranges. Fortunately, the utility of neutral theory also lies in its ability to fail in informative ways (Rosindell et al. 2012).

Our simulations still left much variation in diversity patterns unaccounted for. This could possibly be because our model disregarded historical processes, such as the Cenozoic colonization from mainland Africa and the subsequent formation of neoendemics (Yoder and Nowak 2006; Vences et al. 2009). An alternative consideration is that human‐induced changes in land cover (*e.g.,* Brown and Gurevitch 2004; Kerr et al. 2006) was absent from our simulations. These changes probably have taxon‐specific effects on biodiversity patterns (Brown et al. 2014), which would increase the spatial variation in species richness. These historical and contemporary considerations are supported by comparisons between observed and empirical patterns of species richness (Appendix S4). The neutral model underestimated the species richness of endemic species in the eastern moist forests, which are believed to be centers for the rapid radiation of neoendemics (Wilmé et al. 2006). Similarly, the neutral model also overestimated species richness at the centre of Madagascar, which is now extensively modified by humans (Kerr et al. 2006).

Of course, there are also patterns that obviously demonstrate the limitations of neutral theory. A subtle, yet important, example is the way the neutral model was unable to emulate the increase in community similarity between the most distant quadrats (Fig. 1G and H). The simplest explanation for this is that the quadrats separated by more than 1200 km are at the southern and northern most extremes of Madagascar, which are likely to share several species that do not necessarily occur in the interior (Appendix S4). These species include both migratory and endemic shorebirds that prefer saline and freshwater aquatic habitats along the coastline. Additionally, some species are restricted to low altitudes, which excludes the high‐lying central parts of the island. These cases show that despite being a powerful first approximation, predictions from neutral theory can still be extended by incorporating information on the natural history and ecology of species (Gotelli and McGill 2006; Kalyuzhny et al. 2015).

In conclusion, this study cautions against using simplistic hypotheses, such as the MDE hypothesis, to test the validity of stochastic mechanism in general. Incorporating energetic constrains and long‐distance dispersal into a neutral model allowed us to predict four patterns of Madagascan bird diversity without considering deterministic differences between species. This does not imply that species are indeed equivalent and, therefore, interchangeable. On the contrary, incorporating energy gradients, evolutionary history, and species‐specific habitat requirements into basic stochastic frameworks will improve the accuracy of predictions. Instead, our results demonstrate the importance of zero‐sum dynamics, a long‐distance dispersal in explaining diversity patterns in Madagascar. This justifies conservation efforts that specifically target the maintenance of intact natural habitat and connectivity between protected areas (Wilmé et al. 2006; Kremen et al. 2008).

## Conflict of Interest

None declared.

## Supporting information


**Appendix S1.** The relationship between the total prediction error from the neutral model and the duration of the simulation for all and endemic birds in Madagascar.
**Appendix S2.** Estimates of extinction rates due to ecological drift in the neutral model simulations for all and endemic birds in Madagascar.
**Appendix S3.** The difference between observed and simulated patterns of species richness for all and endemic birds with different geographic extents of occurrence in Madagascar.
**Appendix S4.** The relationship between the number of species in a quadrat and the number of bird units from the neutral model simulation for all and endemic birds in Madagascar.
**Appendix S5.** A nonrandom selection of geographical distribution ranges for Madagascan birds, which illustrate the distribution of species at the most northern and southern extremes of the island.

**Appendix S6.** The data and R‐code to replicate this study.
