# Supplementary material for: Adding energy gradients and long‐distance dispersal to a neutral model improves predictions of Madagascan bird diversity
Source: Ecol Evol. 2016 Sep 7;6(19):6919–29. doi: 10.1002/ece3.2379 (PMC5513242; doi:10.1002/ece3.2379)
Supplement: Supplementary file 1 — Appendix S1. The relationship between the total prediction error from the neutral model and the duration of the simulation for all and endemic birds in Madagascar. Appendix S2. Estimates of extinction rates due to ecological drift in the neutral model simulations for all and endemic birds in Madagascar. Appendix S3. The difference between observed and simulated patterns of species richness for all and endemic birds with different geographic extents of occurrence in Madagascar. Appendix S4. The relationship between the number of species in a quadrat and the number of bird units from the neutral model simulation for all and endemic birds in Madagascar. Appendix S5. A nonrandom selection of geographical distribution ranges for Madagascan birds, which illustrate the distribution of species at the most northern and southern extremes of the island. [file ECE3-6-6919-s001.docx]

**Supplementary Appendix**

**Adding energy gradients and long-distance dispersal to a neutral model improves predictions of Madagascan bird diversity.**

Falko T. Buschke^1,2^*, Luc Brendonck^2^, Bram Vanschoenwinkel^3^

1- Centre for Environmental Management (67), University of the Free State, P.O. Box 339, Bloemfontein 9300, South Africa

2- Laboratory of Aquatic Ecology, Evolution and Conservation, KU Leuven, Ch Deberiotstraat 32, 3000 Leuven, Belgium.

3- Department of Biology, Vrije Universiteit Brussel, Pleinlaan 2, 1050, Brussels, Belgium.

*Corresponding author - falko.buschke@gmail.com

Tel: +27 (0) 51 401 3959

**Appendix S1** The relationship between the total prediction error from the neutral model and the duration of the simulation for all and endemic birds in Madagascar.

**Appendix S2** Estimates of extinction rates due to ecological drift in the neutral model simulations for all and endemic birds in Madagascar.

**Appendix S3** The difference between observed and simulated patterns of species richness for all and endemic birds with different geographic extents of occurrence in Madagascar.

**Appendix S4** The relationship between the number of species in a quadrat and the number of bird units from the neutral model simulation for all and endemic birds in Madagascar.

**Appendix S5** A non-random selection of geographical distribution ranges for Madagascan birds, which illustrate the distribution of species at the most northern and southern extremes of the island.

**Appendix S6** The data and R-code to replicate this study.

**Appendix S1 The relationship between the total prediction error from the neutral model and the duration of the simulation for all and endemic birds in Madagascar.**

As with the majority of stochastic simulation models, our neutral simulations required a burn-in period after initiation. This is illustrated in the rapidly declining total model error at the onset of our simulations (Fig. S1). After approximately 40,000 time-steps in the model, the total model error begins levelling off near the minimum total prediction error.

Since our simulation model did not contain speciation nor immigration from outside of Madagascar, the model error began increasing again after approximately 100,000 time-steps. We expect that this increase is due to the extinction of species due to random ecological drift; both within individual quadrats as well as the entire island. This explanation is supported by increased variation in prediction error between replicated simulations, which is consistent of what would be expected from random drift (i.e. since it is a random process, it will differ between replicates).

The model outcomes presented in the main manuscript were from simulations with 50,000 time-steps. Not only is this the simulation duration where total prediction error is minimised, it is also a duration at which the prediction error is relatively stable and demonstrates the least variation amongst replications of the model (Fig. S1).


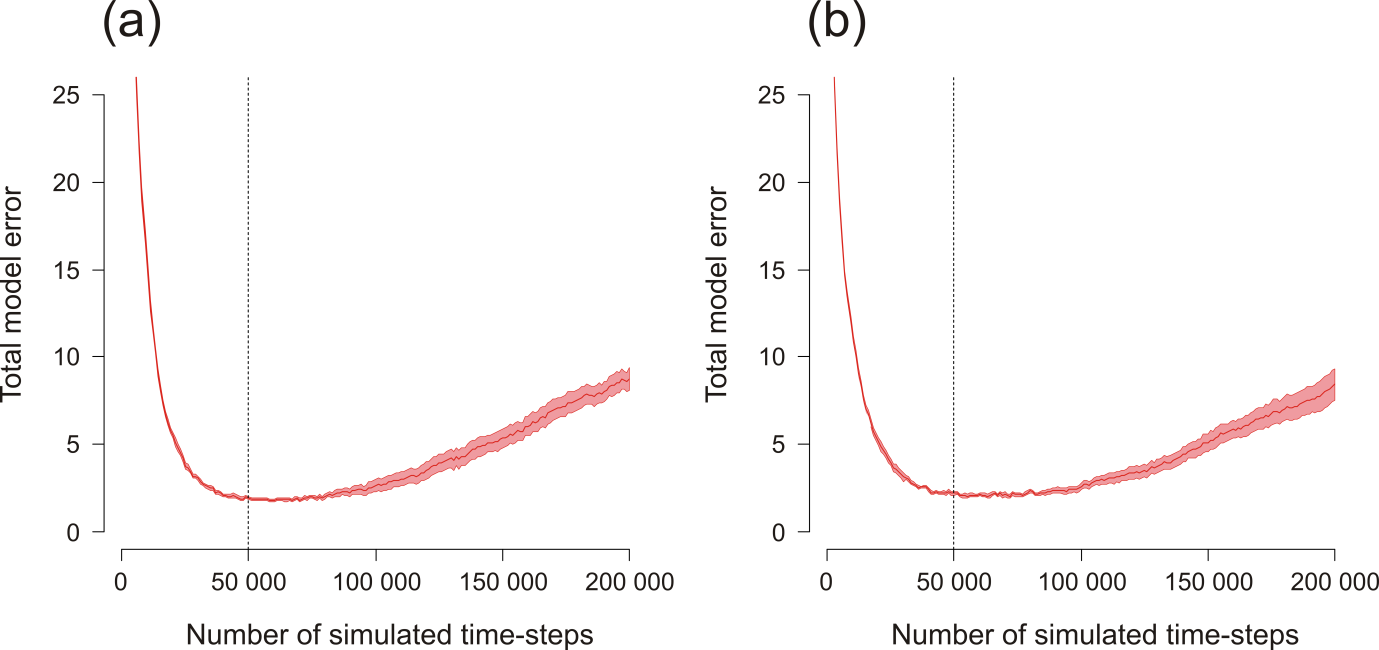


**Figure S1** The change in total model error from neutral simulations as the number of time-steps increase for all (a) and endemic (b) birds in Madagascar. The solid red line represents the mean and the red bands represent the standard error of the total model error from 10 replicated simulations. The vertical dotted line denotes the simulation length used in the main manuscript.

**Appendix S2 Estimates of extinction rates due to ecological drift in the neutral model simulations for all and endemic birds in Madagascar.**

Due to the absence of a speciation parameter in our simulations, species could go extinct due to random ecological drift. Rather than incorporating speciation into our model as an additional free parameter, we assumed that speciation rates must equal extinction rates for the system to reach equilibrium. We, therefore, used extinction rates to infer the speciation rate need to maintain equilibrium.

We ran our simulations as outlined in the main manuscript and recorded the rate at which species went extinct during the 50,000 time-step simulation. We then averaged these values for 10 replicates of the model. Visual inspection suggested that extinction rates were constant through time (i.e. the number of extinct species accumulated linearly through time). Thus, as an estimate of extinction rates, we used the slope of the regression between the number of species still extant in the assemblage and the number of time-steps in the model. However, since every time-step of the model replaced an individual in every one of the quadrats, the extinction rate from the regression represented the extinction rate of the whole island of Madagascar. To calculate the extinction rate of any one quadrat, we simply divided the estimated total extinction rate by the number of quadrats in the simulation.

The slope from a linear regression showed that for all Madagascan bird species, the island-wide extinction rate was 2.65 x 10^-4^ extinctions per time step (Intercept = 222.4; R^2^ = 0.997). This is equivalent to an extinction rate of 9.48 x 10^-7^ in any one quadrat. For endemic birds, the island-wide extinction rate was 1.912 x 10^-4^ extinctions per time step (Intercept = 111.7; R^2^ = 0.998), which is 6.83 x 10^-7^ extinctions in any one quadrat per time-step. It seems sensible that the extinction rates of the whole assemblage are higher than those for only endemic species (because the former encompasses the latter). Moreover, in order to reach equilibrium, extinction in the whole assemblage can potentially be offset by speciation as well as immigration from elsewhere (our simulation would not be able to distinguish between these two events unless we added a fifth free parameter). For endemics species, however, equilibrium can only be reached when speciation rates match extinction rates.

Halley & Iwasa (2011) quantified the time it takes for a the number of species to fall to one-half of its initial value, *T_50_*, and found that this half-life value was related to the area, *A*, of the habitat in which the species occur by the power function *T_50_* = 4.35 x *A*^0.652^. Although, Halley & Iwasa (2011) only examined this relationship up to an area of 1 million hectares, we estimated the *T_50_* value for the whole island of Madagascar (approximately 58.7 million hectares). This calculation estimated the *T_50_* values for Madagascar birds to be 505,631.6 based on the area of the island. However, it is also possible to estimate *T_50_* values from time-series data: $T_{50}= \left[ \frac{S_{t}}{S_{o}-S_{t}} \right].t$, where *S_0_* is the species richness at the start of the time series and *S_t_* is the species richness at time *t.* Substituting the values from our simulation of 50,000 times steps for endemic birds (*S_0_* = 112 and *S_50,000_* = 102), we get a value of *T_50_* = 510,000. This is remarkably close to the average *T_50_* value that would be expected considering the surface area of Madagascar (*T_50_* = 505,631) and suggests that the extinction rates observed in our simulations closely resembled those reported in the literature.

**Appendix S3 The difference between observed and simulated patterns of species richness for all and endemic birds with different geographic extents of occurrence in Madagascar.**

Even though neutral simulations could predict the frequency distribution of species' range sizes, they underestimate the variation in the frequency distribution of species richness. This suggests that the neutral model cannot predict the placement of species ranges. We tested whether this was indeed the case for all species, regardless of their range size. To this end, we calculated the log response ratio ($LRR=ln\left[ \frac{Observed richness}{Simulated richness} \right]$) between observed species richness and simulated species richness (Fig. S2 and S3). The LRR is negative when simulations overestimate species richness, and positive when they underestimate species richness. We first calculated LRR for the all species (whole assemblage in Fig. S2 and endemic species in Fig. S3) and then for sub-assemblages based on range size quartiles.

For both the whole assemblage (Fig. S2) and endemic species (Fig. S3), neutral simulations were able to predict species richness patterns for wide-ranging species accurately. The LRR was narrowly distributed around 0 for the 3^rd^ and 4^th^ range quartiles for both the whole assemblage and the subset of endemic species. For the 3rd quartile, the neutral model overestimated a mid-domain peak in species richness and this overestimation was greater for endemic species.

For narrowly distributed species (1^st^ and 2^nd^ range quartiles), the neutral model was a poor predictor of species richness. For both the whole assemblage (Fig. S2) and the subset of endemic species (Fig. S3), the neutral model overestimated the richness in the western interior of the island and underestimated the richness in the eastern moist forests; especially for endemic species. This patterns in consistent with the hypothesis that Madagascan centres of endemism are due to quaternary aridification, which was more pronounced in river catchments with low-altitude sources; as is the case for the eastern moist forests (Wilmé *et al*., 2006). This implies that radiations of neoendemics after relatively recent (< 4 million years ago) colonisation events (*sensu* Yoder & Nowak, 2006) may account for the poor predictions from our neutral models.


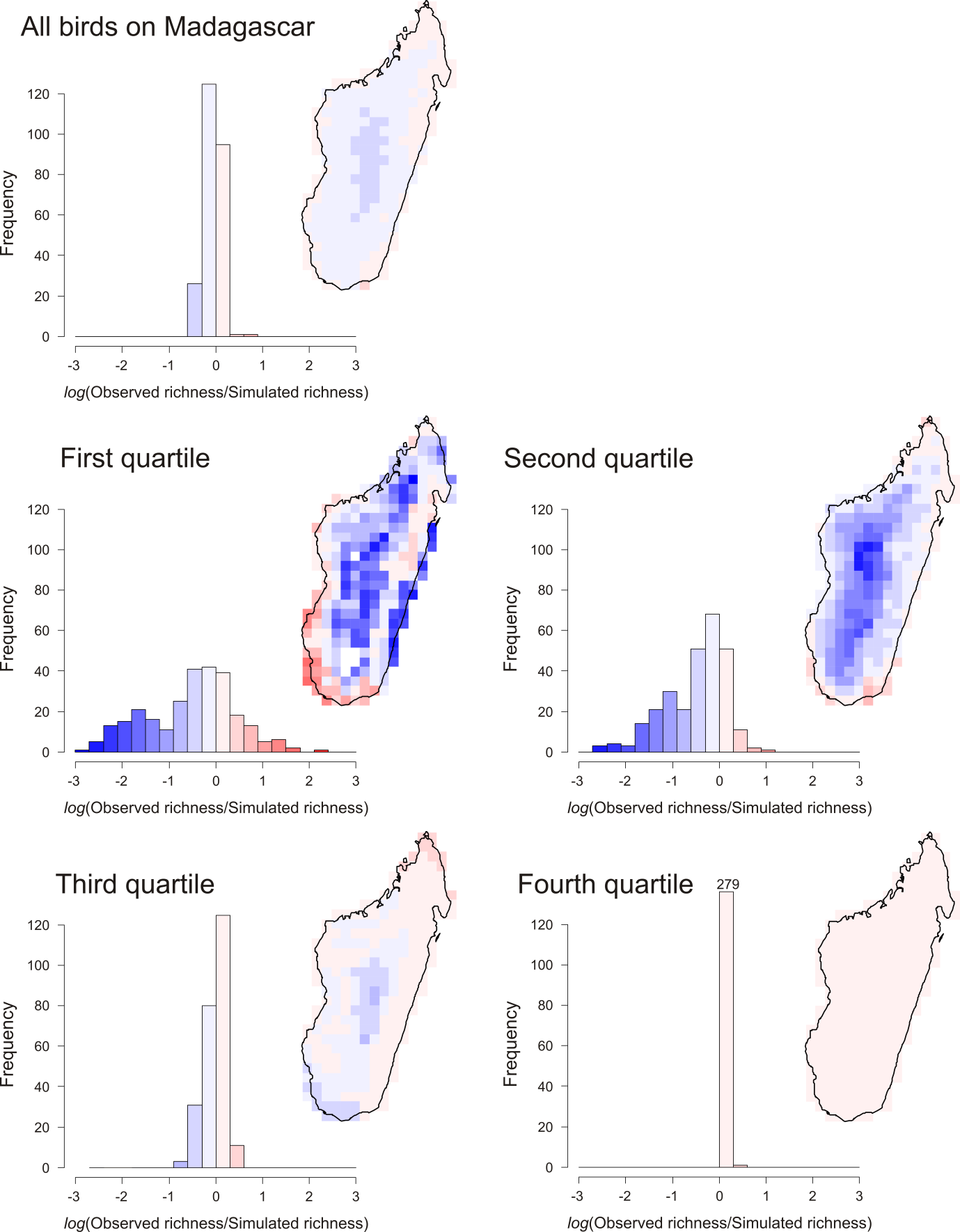


**Figure S2:** The log response ratio between observed and predicted species richness for the whole assemblage of Madagascan birds for different range size quartiles.


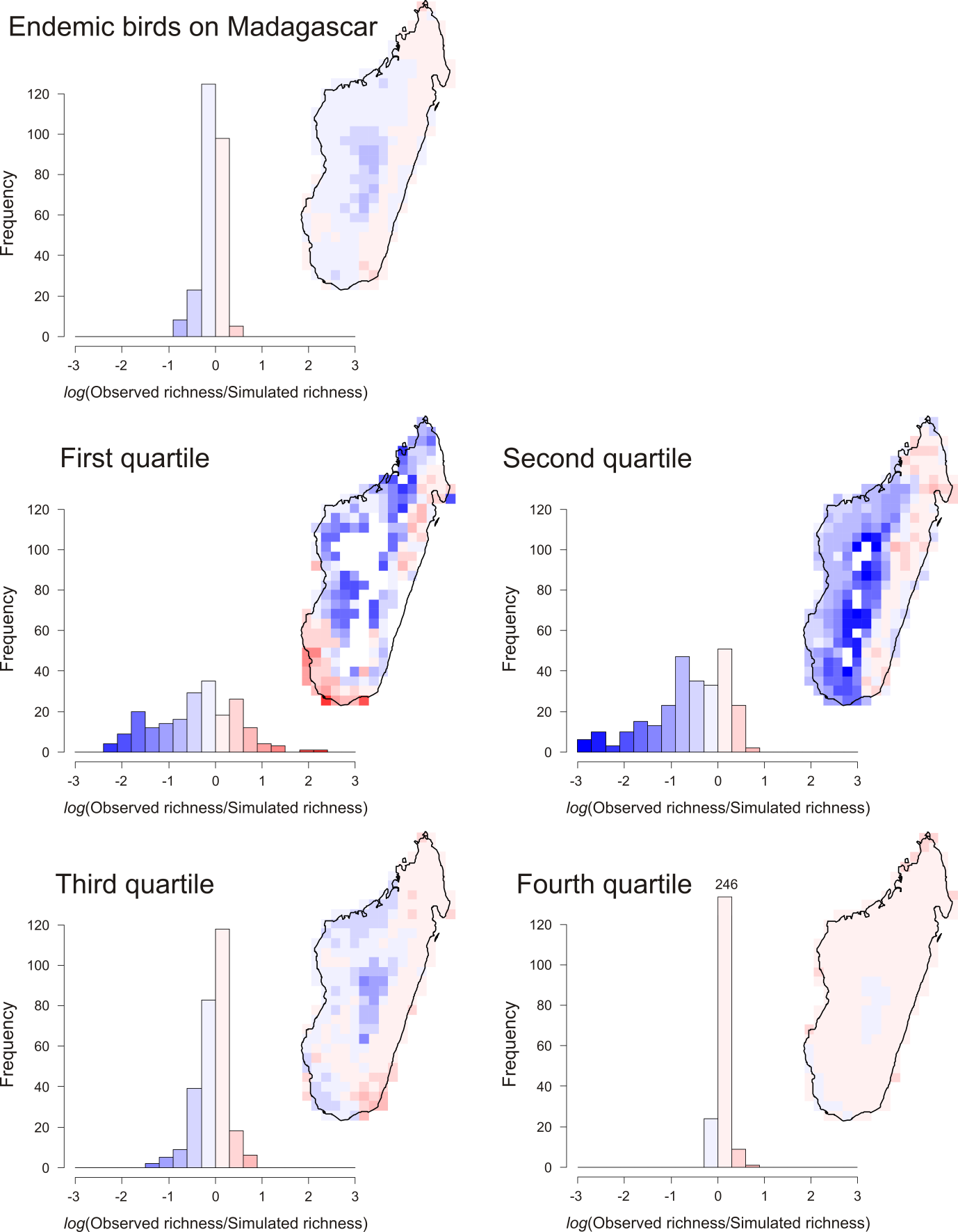


**Figure S3:** The log response ratio between observed and predicted species richness for endemic Madagascan birds for different range size quartiles.

**Appendix S4 The relationship between the number of species in a quadrat and the number of bird units from the neutral model simulation for all and endemic birds in Madagascar**.


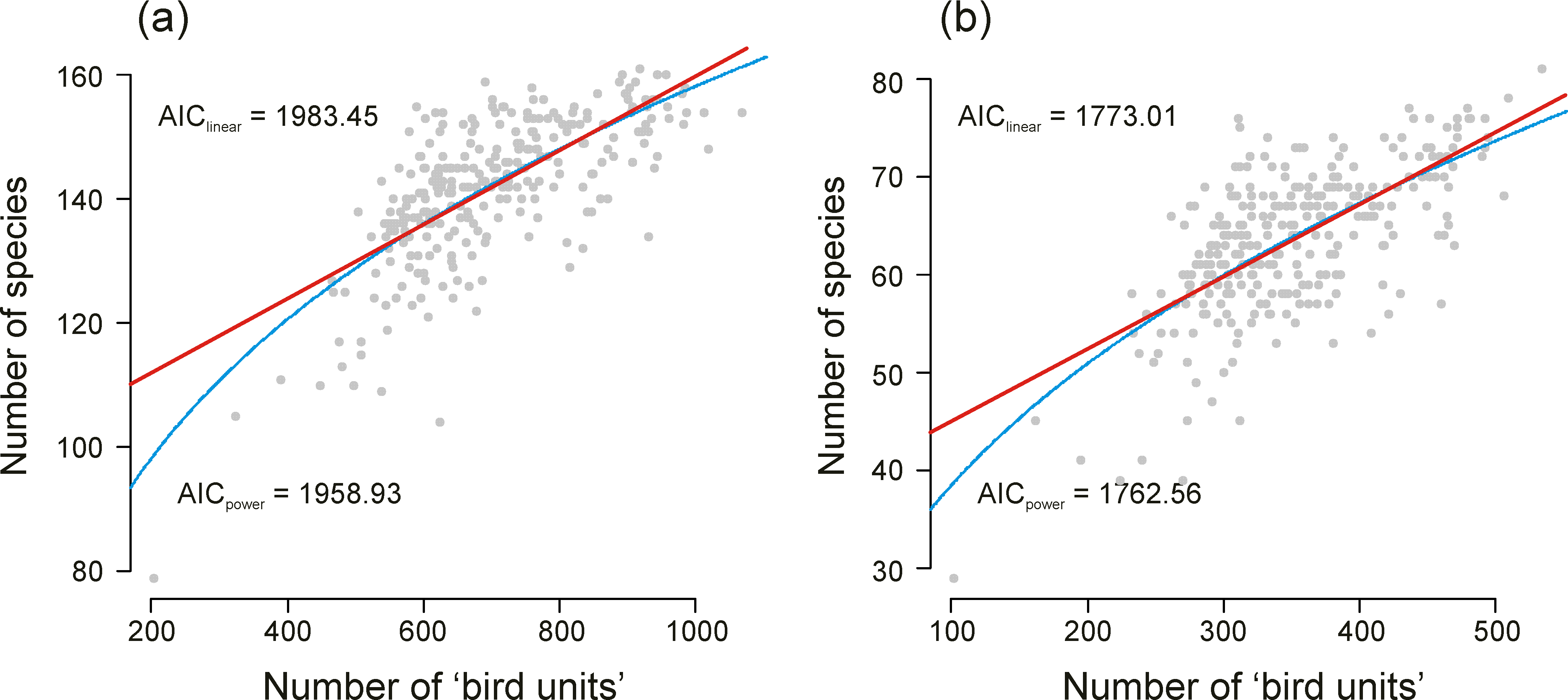


**Figure S4:** The relationship between the number of species in a quadrat and the number of bird units from the neutral model simulation for all (a) and endemic (b) birds in Madagascar. Red lines indicate the best-fitting linear relationship and blue lines indicate the best-fitting power (*y = ɑ.x^β^*) relationship. AIC values denote the Akaike's Information Criterion for the linear and power functions.

**Appendix S5 A non-random selection of geographical distribution ranges for Madagascan birds, which illustrate the distribution of species at the most northern and southern extremes of the island.**

Even though neutral theory successfully predicted the distance-decay of Jaccard similarity, there where two general exceptions. The first exception was the underestimation of similarity at short distances (< 200 km). While this may be a limitation of neutral theory, we should not discount the possibility that empirical patterns based on extent of occurrence (EOO) data overestimate Jaccard similarity at short distances. EOO data are derived from the interpolation of marginal occurrences from several years' worth of inventory data and may possibly overestimate the cohesiveness of species distributions and, therefore, the compositional similarity at short distances.

The second exception was the propensity of the neutral model to underestimate similarity at exceptionally large geographic distances (> 1200 km). In simple terms, this means that the species composition at the most northern- and southern-extremes of the island (which are approximately 1500 km apart) are more similar than expect from neutral processes. This could be due to the distribution of migratory shorebirds, which are restricted to Madagascan coastlines (Fig. S5.). Examples of these shorebirds are the ruddy turnstone (*Arenaria interpres*), black-winged stilt (*Himantopus himantopus*) and the grey plover (*Pluvialis squatorola*) (Fig. S5). Similarly, endemics, such as the Madagascar heron (*Ardea humbloti*) and the Madagascar sacred ibis (*Threskiornis bernei*), also prefer saline and freshwater habitats along the entire western coastline, while other widespread endemics, such as the white-browed hawk-owl (*Ninox superciliaris*), are restricted to altitudes less than 800 m (Fig. S5). In these instances, the same bird species will occur in habitats that are geographically far apart, and the neutral model would be unable to recreate these patterns. These habitat effects do not invalidate the neutral theory approach, but rather illustrate is usefulness as a null model because the effect of habitat was informed by divergence from neutral expectations.


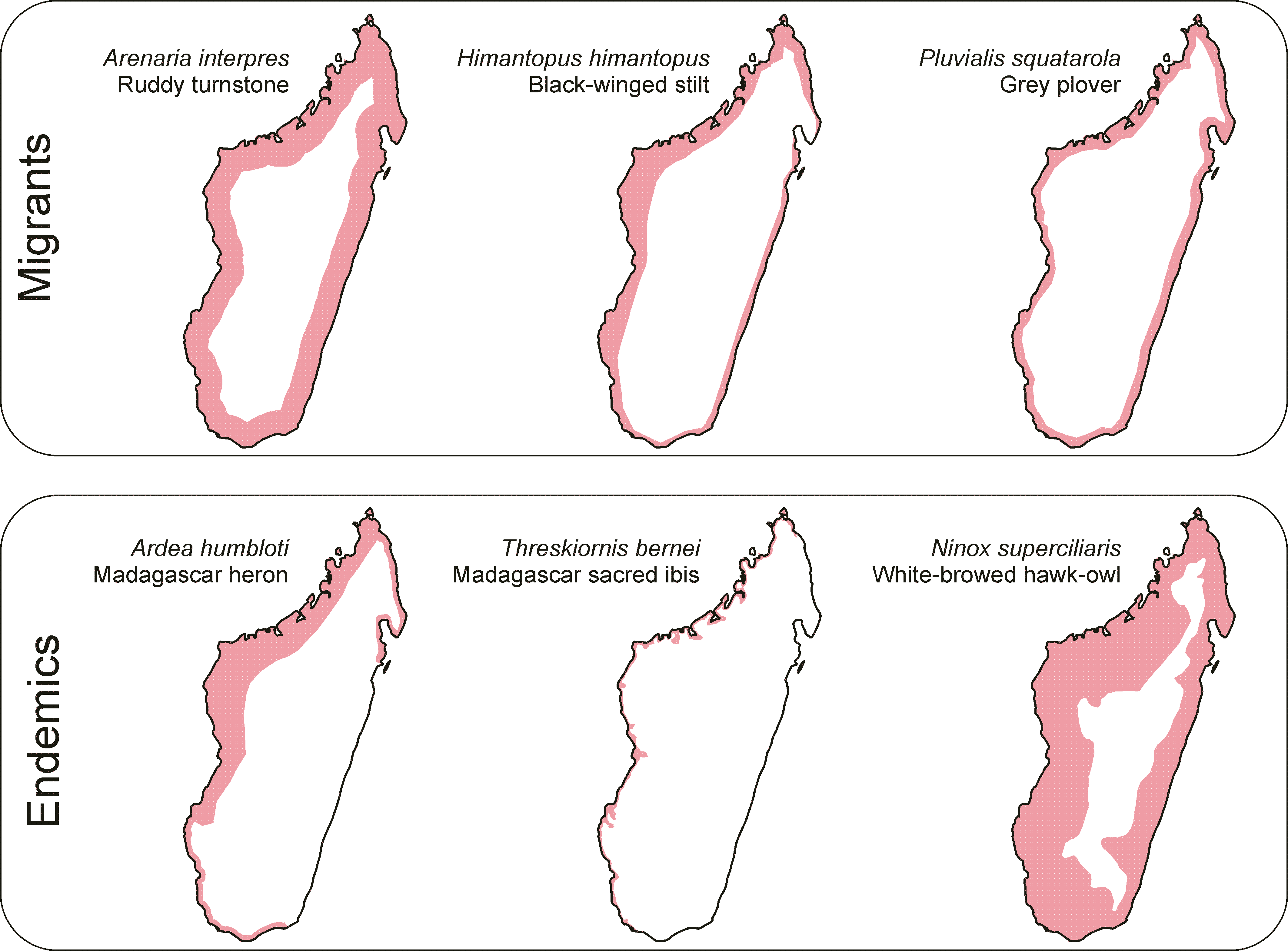


**Figure S5** A non-random selection of geographical distribution ranges for Madagascan birds.
